# Myocardial Extracellular Volume Fraction Measured by Cardiac Magnetic Resonance Imaging Negatively Correlates With Cardiomyocyte Breadth in a Healthy Porcine Model

**DOI:** 10.3389/fcvm.2022.791963

**Published:** 2022-03-17

**Authors:** Shi-Jun Zhang, Di Chang, Ji-Yang Jin, Ya-Ling Wang, Lin Wang, Yuan-Cheng Wang, Zhen Wang, Shenghong Ju

**Affiliations:** ^1^Department of Radiology, Zhongda Hospital, Jiangsu Key Laboratory of Molecular and Functional Imaging, Medical School of Southeast University, Nanjing, China; ^2^Department of Anesthesiology, Zhongda Hospital, Medical School of Southeast University, Nanjing, China

**Keywords:** extracellular volume fraction, cardiac magnetic resonance imaging, T1 mapping, myocardium, cardiomyocyte breadth

## Abstract

**Background:**

The extracellular volume fraction (ECV) derived from cardiac magnetic resonance imaging (MRI) is extensively used to evaluate myocardial fibrosis. However, due to the limited histological verification in healthy individuals, it remains unclear whether the size of cardiomyocytes may play a potential role in the physiological changes of ECV. The aim of this study was to examine the association between cardiomyocyte size and myocardial ECV by using a healthy porcine model.

**Methods:**

Sixteen domestic healthy pigs were anesthetized and underwent cardiac MRI with mechanical controlled breathing. Intravenous contrast medium was introduced at a dose of 0.2–0.25 mmol/kg. The interventricular septum ECV was calculated using an established MRI procedure, which was based on the pre- and post-contrast T1 values of the heart and individual blood hematocrit. The cardiomyocyte breadth (CmyB) in cross section was measured by hematoxylin and eosin staining to reflect the cardiomyocyte size.

**Results:**

Data were successfully acquired from 14 pigs. The CmyB was obtained from the myocardial tissues corresponding to the region of interest on cardiac MRI. The mean ± SD of the ECV was 0.253 ± 0.043, and the mean ± SD of the CmyB was 10.02 ± 0.84 μm. The ECV exhibited a negative correlation with the CmyB (*r* = −0.729, *p* = 0.003).

**Conclusion:**

The myocardial ECV detected by cardiac MRI is negatively correlated with the CmyB in healthy pigs, demonstrating that the size of cardiomyocytes is potentially associated with the ECV under physiological conditions.

## Introduction

The *in vivo* non-invasive calculation of the myocardial extracellular volume fraction (ECV) by cardiac magnetic resonance imaging (MRI) is useful for estimating myocardial fibrosis (1–5). Previous studies have shown that the ECV in normal myocardium varies greatly (6–10), suggesting that ECV carries unknown information with physiological relevance, and a broad physiological distribution of the ECV limits the specificity and sensitivity of ECV as a fibrosis index. In-depth investigations of the ECV under physiological conditions will contribute to a further understanding of ECV, expediting a better utilization of this indicator (11–14).

Recent studies have proposed that other than reflecting a relative value of the volumes of the extracellular space, ECV may also be associated with the volume of cardiomyocytes (15–17), and a change in either the volume of the extracellular space or the volume of the intracellular space may affect the ECV. In other words, the difference of ECV in a unit volume may be related to both the content of extracellular interstitial volume and the cardiomyocyte size. However, to date it is unclear whether the differences in ECV under physiological conditions are related to a single factor of the cardiomyocyte size.

So far, the investigation of the relationship between ECV and histopathological measurements in healthy individuals has been largely restricted because it is difficult to perform biopsy in healthy people. Pathological analysis has shown that cardiomyocytes have an approximately cylindrical shape with a length that is significantly greater than the breadth, and a change in the breadth may better reflect a change in the volume of the cardiomyocytes as described in a previous study (18). Thus, the cardiomyocyte breadth (CmyB) is better to be applied to quantify the size of cardiomyocytes. In this study, we aimed to investigate whether the size of cardiomyocytes may play a potential role in the physiological changes of ECV, and the correlation of the ECV and the CmyB was determined *via in vivo* cardiac MRI and *ex vivo* myocardial pathology by using a healthy porcine model.

## Materials and Methods

### Animal Preparation

The animal experiments in this study were approved by the Institutional Animal Use and Care Committee of the Southeast University. This work was carried out in compliance with the ARRIVE guidelines (19), and all experiments were carried out blindly and animals were randomly selected from pigs with 8 to 12 weeks old. A total of 16 domestic healthy pigs (including eight males and eight females, 8 to 11 weeks old, weighing 13.6–30.0 kg, with an average weight of 24 kg) were intramuscularly injected with ketamine (4–5 mg/kg) before surgery. There were no specific inclusion and exclusion criteria for animals. An intravenous indwelling needle connected to a catheter was placed in the ear to allow venous access. Venous blood (2–4 mL) was collected for routine hematology examinations, and the hematocrit was calculated. The anesthesia was maintained by the intravenous infusion of sodium pentobarbital, with an administration rate of 0.1 mg/kg/min. A regular 6.0 mm or 6.5 mm endotracheal tube was used for the endotracheal intubation of the animals, and a specialized anesthesia machine was used to maintain and control the breathing of the pigs. The animals were fixed in the supine position and a tail-first orientation using a special bracket; the chest was covered with a coil used for cardiac MRI, and a sensor cushion used to monitor respiration was placed between the coil and the abdominal wall. The hair on tail of the animal was removed, and the tail was clamped with a dedicated fingertip-type pulse and oxygen detector to detect the pulse rate for triggering scanning during cardiac MRI.

### Cardiac Magnetic Resonance Imaging

Cardiac MRI was performed using a 3.0 Tesla clinical MRI system (MAGNETOM Verio, Siemens AG Healthcare Sector, Erlangen, Germany). A six-channel surface array coil and a six-channel spine coil were used for signal acquisition. The short axis plane at the mid-papillary muscle level was selected as the scanning dimension, and a modified Look-Locker inversion-recovery (MOLLI) sequence was used for T1 mapping. The T1 map before the enhancement was obtained with a MOLLI4(7)3 acquisition sequence, and the T1 maps at 1 to 60 min after the enhancement were obtained with repeated MOLLI4(3)3 acquisition sequences as we previously reported (20). The interval between two adjacent acquisitions after contrast enhancement was gradually increased from 0.5 to 5 min. An appropriate delay time for the pulse trigger approach was set in the system so that the acquisition window occurred during the mid-late diastolic phase, and the frequency of the trigger pulse was set to every two beats (approximately 55–70 beats/min, which is equivalent to the heart rate of a human). The other MOLLI parameters were set as follows: an in-plane resolution of 1.8 mm × 2.2 mm, a matrix size of 192 × 78, slice thickness of 8 mm, balanced steady-state free precession readout, repetition time = 2.5 ms, echo time = 1.1 ms, flip angle of 35°, integrated parallel acquisition technology with an acceleration factor of two, and a 6/8 partial Fourier acquisition.

The enhanced scanning procedure was performed with gadolinium (Magnevist, 0.5 mmol/mL, Bayer AG, Leverkusen, Germany) at the dose of 0.2–0.25 mmol/kg, which was rapidly injected through the ear vein (approximately 0.5 mL/s), followed by flushing the vein with 5 mL saline. The region of interest (ROI) was carefully selected from the interventricular septum, avoiding the cardiac-blood border zone, for calculation of the ECV. The ECV was calculated using an established equation: ECV = λ × (1 – hematocrit), where λ was separately calculated in each animal using a multi-point regression method and was equal to the slope of the linear regression for the individual myocardial R1 value plotted against the left ventricular blood R1 value in the equilibrium state, as described in our previous study (20), and the equilibrium state of the contrast agent between the blood and tissue was determined according to established methods. The cardiac MR analyses were performed by 2 radiologist (Z.S.J., 10 years of experience with cardiac MRI, and C.D., 8 years of experience with cardiac MRI) who were blinded to the animals.

### Histology

The animals were euthanized after the cardiac MRI procedure by an intravenous injection of a high concentration of potassium chloride. The heart was completely separated, and each heart chamber was rinsed with saline. The left and right ventricles were filled with 3% agarose gel, and the heart was embedded. The structure and volume of the left and right ventricles were retained. Sectioning was performed using a specialized slicer with a thickness of 6 mm and carefully matching the corresponding scan layer of the cardiac MRI. In the resulting slice, the obtained interventricular septum tissue was divided into two sections consisting of the anteroseptal region and the inferoseptal region. After removing the junction of the left and right ventricles, the specimen was fixed in formalin solution for 48 h. The following biopsy work was performed by a pathological technician (W.Y.L., 8 years of experience with pathological staining) who was blind to the cardiac MRI study. The tissue was selected, and the orientation of the tissue block was labeled, followed by paraffin sectioning with the slice thickness of 4 μm. The slicing dimension was perpendicular to the annular fibers of the midmyocardium.

Conventional hematoxylin and eosin (H&E) staining were performed. A cross-section of the midmyocardium fibers was viewed under a 200 × optical microscope (Olympus), and the boundary of the inner and outer layers was identified. The midmyocardium fibers were divided into four equal areas in the direction of the endocardium to the epicardium. Under 400 × magnification, a random field was selected from each area (356 μm × 266 μm; 100–200 cells in each area), and the images of the fields of view were captured and saved. Thus, images of the cross section of the midmyocardium fibers from eight interventricular septa of each animal were obtained at 400 × magnification, and more than one thousand cells were measured in each animal. Off-line measurements and analyses were performed using dedicated software (Image-Pro Plus 6.0) by 2 blinded readers (W.Y.L., 8 years of experience with pathological staining, and W.L., 6 years of experience with pathological staining). The cross-section of each cardiomyocyte with an intact and approximately oval, blue-stained nucleus in each center was included in the analysis. The cross-sections were approximately oval-shaped, and the shorter diameter of this oval was recorded as the CmyB (18).

### Interobserver and Intraobserver Variability

A total of 28 pathological slices was acquired from 14 pigs with two slices per animal. Then, a subset of 12 slices was randomly selected to measure the interobserver and intraobserver variability. To derive the interobserver variability, two blinded investigators were asked to choose the fields of view and perform these measurements by themselves. To assess the intraobserver variability, one blinded investigator measured the same slices again on a separate day and performed these measurements twice.

### Statistical Analysis

Normal distributions of variables were evaluated before analysis using the Shapiro–Wilk test. The values of ECV and CmyB were processed as continuous variable of normal distribution according to previous studies and were reported as means ± standard deviation (SD) (1, 4–9, 18). The relationship between the ECV and CmyB was investigated using Pearson’s simple correlation analysis. Interobserver and intraobserver agreement of the histopathologic diagnosis were calculated by use of κ statistics. Calculations were performed by use of the Statistical Package for Social Sciences software (SPSS). A *p*-value less than 0.05 was considered a statistically significant difference.

## Results

### Mortality Rate and Animal Characteristics

One of the 16 pigs died of suffocation during the cardiac MRI procedure due to a failure of endotracheal intubation (Animal number 4) with a mortality rate of 6.25%, and another animal experienced enhanced scan failure due to severe extravasation of the contrast agent caused by poor care for the ear vein access (Animal number 16). The data of 14 pigs were included in the final analysis, and detailed gender, age, heart rate, systolic blood pressure, hematocrit, body weight, ECV and CmyB values were acquired (Table 1). Dynamic equilibrium state was established in all 14 animals within 8 min.

**TABLE 1:** Animal characteristics and individual data.

No.	Gender	Age (week)	Heart rate	Systolic BP (mmHg)	Hematocrit	Body weight (kg)	ECV	CmyB (μ m)
(1)	M	10	136	109	0.30	26.0	0.272	10.87 ± 1.55
(2)	F	8	116	97	0.23	13.6	0.335	8.64 ± 1.56
(3)	F	9	133	91	0.21	20.0	0.325	9.06 ± 1.48
(4)	F	9	129	112	0.34	20.5		
(5)	M	10	120	106	0.31	26.0	0.292	8.96 ± 1.38
(6)	F	11	140	98	0.26	27.5	0.255	10.55 ± 1.55
(7)	M	10	119	101	0.35	23.0	0.252	9.33 ± 1.24
(8)	M	10	134	116	0.34	25.5	0.272	9.91 ± 1.50
(9)	M	9	109	113	0.37	25.0	0.202	10.23 ± 1.70
(10)	M	10	112	96	0.34	25.0	0.240	10.18 ± 1.77
(11)	M	10	110	117	0.36	25.0	0.219	10.75 ± 1.15
(12)	M	9	112	107	0.31	25.0	0.241	10.64 ± 1.41
(13)	F	12	126	92	0.29	30.0	0.232	9.28 ± 1.37
(14)	F	12	122	94	0.34	29.0	0.217	10.39 ± 1.35
(15)	F	10	134	105	0.34	23.0	0.190	11.48 ± 2.07
(16)	F	11	118	93		25.0		

*The CmyB was calculated as the mean ± SD. ECV indicates extracellular volume fraction; CmyB, cardiomyocyte breadth; SD, standard deviation.*

### T1 Mapping and Contrast Agent Distribution Coefficient (λ)

**FIGURE 1:**
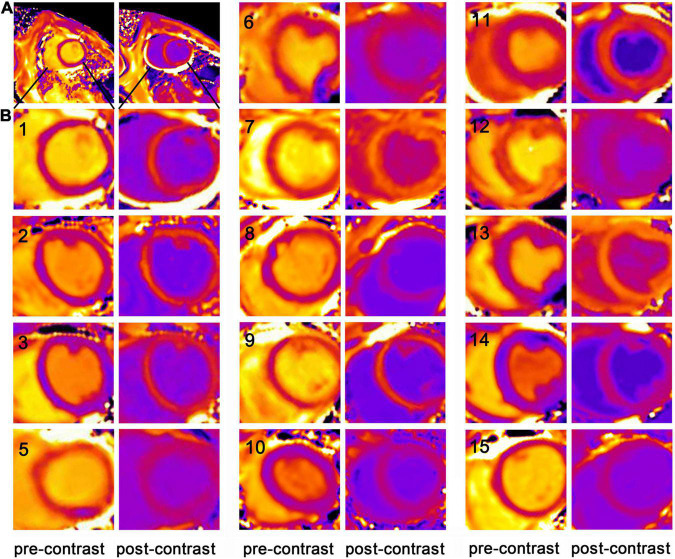
The short-axis T1 maps of the porcine model. **(A)** An example of the short-axis T1 maps at the mid-papillary muscle level pre- and post-contrast in the healthy porcine model (Animal number 1). Only one representative post-contrast T1 map was chosen from multiple post-contrast time points and displayed here. **(B)** As artifacts occasionally appeared on the free wall of the left ventricle, but no artifact was detected in the ventricular septum, the magnified images focusing on the ventricular septum at the mid-papillary muscle level pre- and post-contrast in the 14 healthy pigs were displayed (with each animal number listed in the images of pre-contrast T1 maps). Animal number 4 died and Animal number 16 experienced enhanced scan failure. T1 values were measured from the ventricular septum and the left ventricular blood pool.

We acquired the short-axis T1 maps at the mid-papillary muscle level pre- and post-contrast of the animals. Although artifacts occasionally appeared on the free wall of the left ventricle, there was no artifact in the ventricular septum (Figure 1A). Thus, T1 values were measured from the ventricular septum and the left ventricular blood pool in the 14 healthy pigs (Figure 1B). Then, the myocardial R1 value and the left ventricular blood R1 value were calculated as the reciprocal of the T1 values.

**FIGURE 2:**
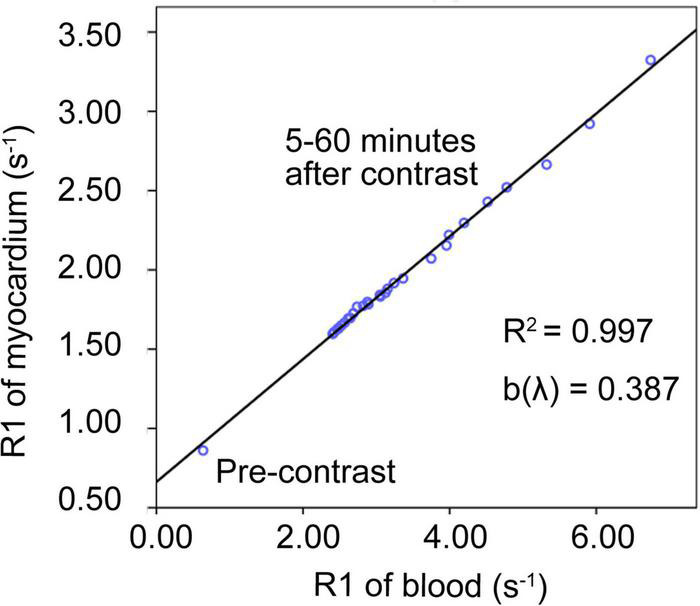
Contrast agent distribution coefficient (λ). An example of the linear regression relationship between the myocardial R1 value and the blood R1 value before (0 min) and after enhancement (5–60 min) in one of the pigs. The data of pre-contrast shows the smallest R1 of myocardium and R1 of blood, while the data of 5 min shows the largest R1 of myocardium and R1 of blood. Within the observation time window between 5 and 60 min after the injection of the contrast agent, the R1 values of the ventricular septum and the left ventricular blood recover proportionally and become closer to the pre-contrast level from 5 to 60 min. The scatter plot demonstrates the validity of the linear regression model. The slope of the line was defined as the distribution coefficient of the contrast agent (λ).

The relationship between the myocardial R1 value and the blood R1 value pre-contrast (0 min) and post-contrast (5–60 min) in each pig was illustrated (Figure 2), and the interval between two adjacent acquisitions after contrast enhancement was gradually increased from 0.5 to 5 min. The R1 data of 1–5 min after the enhancement were not listed in the figure because the contrast agent distribution did not achieve blood-myocardial contrast equilibrium until 5 min after the enhancement. The data of pre-contrast shows the smallest R1 of myocardium and R1 of blood, while the data of 5 min shows the largest R1 of myocardium and R1 of blood. Within the observation time window between 5 and 60 min after the injection of the contrast agent, the R1 values of the ventricular septum and the left ventricular blood recover proportionally and become closer to the pre-contrast level from 5 to 60 min. The scatter plot demonstrates the validity of the linear regression model, and the slope of the regression equation (b value) represents the contrast agent distribution coefficient (λ). This figure confirmed that the method and experimental condition we used to calculate λ and ECV are feasible.

### Cardiomyocyte Breadth

**FIGURE 3:**
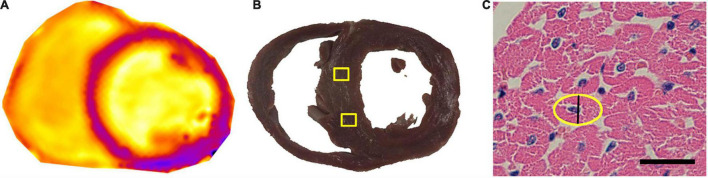
Pathological ROIs corresponding to the T1 map, and CmyB measurement in H&E staining. **(A)** T1 map of the heart before enhancement. **(B)** The thickness of the gross pathological slice was 6 mm, and the yellow boxes indicate the ROIs of sampling areas corresponding to the T1 map. **(C)** Myocardial sections of H&E staining were observed at 400 × magnification, bar = 20 μm; the yellow oval indicates the contour of the cross-section of the cardiomyocyte, and the black line shows the CmyB (10.2 μm). ROI indicates region of interest; CmyB, cardiomyocyte breadth; H&E, hematoxylin and eosin.

Myocardial sections with a thickness of 6 mm carefully matches the corresponding scan layer on cardiac MRI (Figures 3A,B). The H&E staining of cardiomyocytes in cross-section were acquired, and the shorter diameter of this oval was recorded as the CmyB (Figure 3C). The values of CmyB in each pig were detected (Table 1), and the average value of CmyB in all the 14 pigs was 10.02 ± 0.84 μm. The interobserver and intraobserver reliabilities of histopathologic assessment were high (κ values of 0.892 and 0.915, respectively).

### Correlation Between the Extracellular Volume Fraction and Cardiomyocyte Breadth

**FIGURE 4:**
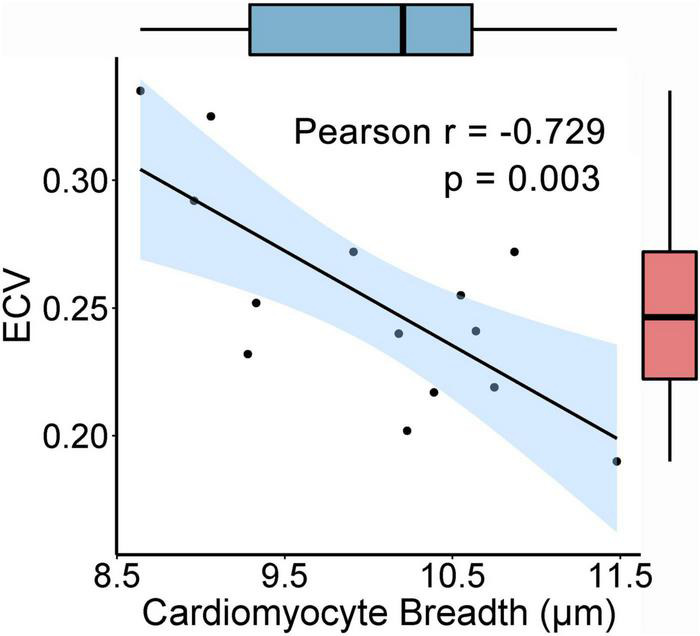
Correlation between the ECV and the CmyB. The ECV is negatively correlated with the CmyB (*r* = −0.729, *p* = 0.003, *n* = 14). 95% confidence interval of the correlation coefficient r values is presented as the light blue area, and the box plots showed that there were no potential outliers of the ECV and CmyB. ECV indicates extracellular volume fraction; CmyB, cardiomyocyte breadth.

The values of ECV in each pig were detected (Table 1), and the average ECV for all animals was 0.253 ± 0.043. In the histological analysis, the number of verified cells counted per field was approximately 100–160, and at least 1,000 cells per animal were included in the statistical analysis for assessing the CmyB. Pearson’s simple correlation analysis showed that the ECV exhibited a negative correlation with the CmyB in this healthy porcine model (*r* = −0.729, *p* = 0.003) (Figure 4).

## Discussion

Extracellular volume fraction has been widely used in the field of cardiovascular imaging, showing great potential due to its unique properties in the non-invasive evaluation of subtle abnormalities such as diffuse fibrosis and post-infarct remodeling of remote myocardium in cardiovascular diseases (21–23). However, the association between the ECV and the size of cardiomyocytes is still unclear. The physiological modeling of the myocardium during growth and development can cause alterations in the ratio of the intracellular volume and the extracellular volume. An increase in the CmyB under physiological conditions induced by inter-subject variation will result in a decrease in the ECV detected by cardiac MRI in healthy myocardium, which is potentially due to genetic and environmental factors, suggesting that an increase in the volume of cardiomyocytes is not always accompanied by a proportional increase in the volume of extracellular space. In this study, the correlation of myocardial ECV and histological breadth of cardiomyocytes under physiological condition was analyzed *via* large animal experiments of pigs. To our knowledge, this is the first study that uses a healthy porcine model to study the relationship between the ECV and the CmyB by using *in vivo* cardiac MRI and *ex vivo* myocardial pathology.

Cardiac ECV is a quantitative value that could reflect various myocardial tissue characterization, especially myocardial fibrosis (24–34). Histological verification of the ECV in reflecting collagen deposition; however, has only been reported by a small number of qualified centers, and most later studies have followed their data and used the ECV as a direct indicator of myocardial fibrosis (6, 26). In most relevant studies, changes in the ECV were explained using a single factor, the volume of the extracellular space, and fibrosis was considered the most common cause of alterations in ECV (26–33). However, although elevated ECV in the late stage of diseases are mainly due to interstitial fibrosis in certain diseases, the impact of cardiomyocyte size on ECV cannot be ignored, especially under physiological conditions and in early stages of diseases, for example cases of cardiac overloaded pressure such as aortic stenosis (35–37) and hypertension (38, 39).

Previous studies by our research team have shown that the variations of ECV detected by cardiac MRI in healthy individuals follow certain rules, which indicates that the ECV and cardiomyocytes are intrinsically linked (20). In the early stage of hypertension, the ECV decreased rather than increased during the adaptive growth process of the heart, suggesting that although myocardial fibrosis elevates the ECV, cardiac hypertrophy can also lead to a decrease in the ECV (24). This finding prompted us to propose a hypothesis that the size of cardiomyocytes may be one cause of the physiological changes of ECV. To verify this hypothesis, the present study calculated the cardiac ECV in pigs using a multi-point regression method and confirmed the dynamic equilibrium of the myocardial R1 and blood R1 values. Due to the endotracheal intubation with assisted ventilation, a relatively ideal experimental state was achieved in animals, and acquisition of data at more time points could be conducted after the enhancement; thus, the regression method with multi-point measurements was more stable and reliable.

So far, research on the size of cardiomyocytes has been largely limited because it is difficult to acquire biopsy in humans, especially in healthy people. Under physiological conditions, the size of cardiomyocytes may be related to several factors such as age, height, weight, hormone levels and different volumes of exercise, some of which have already been confirmed by cardiac MRI or myocardial autopsy (40–44). In addition, a small number of research teams have revealed that the ECV calculated by cardiac MRI may be associated with physiological factors such as age, gender and exercise (45–48). However, regardless of the potential relationship between these physiological factors and CmyB or ECV, there is currently no research focusing on characterizing the association between the CmyB and the ECV, and how a single CmyB may affect the ECV from a pathological perspective in healthy individuals.

In the current study, we used a healthy porcine model to explore the relationship between the ECV and the CmyB, and a significant negative correlation was observed between the ECV detected by cardiac MRI and the CmyB detected by pathological analysis. These results highlight the importance of CmyB to be considered as a direct influencing factor of ECV, and emphasize the necessity to explain ECV in terms of both the volume of extracellular space and the volume of cardiomyocytes. Moreover, our research may help explain why ECV is affected by the above-mentioned physiological factors (such as age, gender, weight, and exercise) in the absence of diseases that can cause changes in the extracellular volume (such as collagen deposition and myocardial fibrosis). It is probably that compared with the volume of extracellular space, the volume of cardiomyocytes may have a greater impact on ECV under physiological conditions. Besides, it is potentially that CmyB might also be one of the influencing factors of ECV in diseased individuals, especially patients with altered sizes of cardiomyocytes, such as hypertrophic cardiomyopathy and Anderson-Fabry disease (49, 50), which is currently unclear and needs to be confirmed by further research.

To date, no simple and practical method has been developed to measure the size of cardiomyocyte in three dimensions in a slice because the intercalated disk structure of a cardiomyocyte may not be fully displayed in one slice, and it is difficult to distinguish the boundaries of two adjacent cardiomyocytes. In addition, because cardiomyocytes show some degree of localized distortion, it is not practical to obtain a standard section of the major axis or minor axis. If the major axis of the cardiomyocyte is not precisely vertical in the section, the resulting short-axis section will be oblong. When the tilt angle is increased, the longer diameter of the oblong axis will be further increased, while the shorter diameter will remain unchanged. Therefore, measurement of the shorter diameter can be more stable and accurate in describing the CmyB, as mentioned in a previous study (18). Thus, here we used the CmyB to reflect the size of cardiomyocytes, and to detect the association between the CmyB and the ECV.

## Limitations

Some limitations were present in this study. Firstly, the study had a small sample size. Due to the limited number of samples, further stratified analyses were not implemented; for example, it has not been determined whether gender influences the results. Despite that, we have already uncovered a negative correlation between the ECV and the CmyB, and confirmed the importance of CmyB in ECV interpretation. Further experiments depending on a larger sample size will improve our results. Secondly, an artifact occasionally occurred in the free wall that might affect the calculation of the ECV in this region. Thus, the analysis of the ECV and the myocardial histology was restricted to the interventricular septum to avoid artifacts. Finally, formalin fixation can cause cell shrinkage (51) and hyperkalemia (52) can cause cell swelling in tissue preparation, which may have altered the myocyte size. But as the hearts of all pigs acquired the same degree of formalin fixation for 48 h, and due to the relative comparisons between the CmyB and the ECV, the degree of myocyte shrinkage in histologic measurements may be ignored (53). Similarly, although potassium chloride will lead to myocyte swelling, due to the same hyperkalemic treatment for all the animals, it had little impact on the correlation analysis as measured in this study.

## Conclusion

Our study demonstrated a significant negative correlation between the ECV and the CmyB in a healthy porcine model, suggesting that an increase in the CmyB under physiological conditions can lead to a decrease in the ECV as detected by *in vivo* cardiac MRI. Although with limited sample size, we hypothesize that a change in the volume of cardiomyocytes may be associated with a change in the ECV, and the difference in the size of cardiomyocytes may exert a key role in the physiological alterations of the ECV. These observations are novel and potentially important for clinical application, especially in understanding the inter-subject variations of the ECV in healthy myocardium under physiological state, and might potentially explain early pathological changes of cardiovascular diseases observed *via* ECV through cardiac MRI. Moreover, the current results strongly suggest that in addition to the extracellular space, we should take CmyB into consideration when interpreting the ECV detected by cardiac MRI in healthy individuals, and potentially in diseased individuals, especially patients with altered sizes of cardiomyocytes, such as hypertrophic cardiomyopathy.

## Data Availability Statement

The original contributions presented in the study are included in the article/supplementary material, further inquiries can be directed to the corresponding author.

## Ethics Statement

The animal study was reviewed and approved by Institutional Animal Use and Care Committee of the Southeast University.

## Author Contributions

S-JZ, DC, and SJ are the guarantors of integrity of the entire study and participated in the design and coordination of the study. S-JZ, DC, and J-YJ conceived the study, and acquired and analyzed the cardiac MR data. J-YJ, Y-LW, and LW performed the pathological analyses. DC, Y-LW, LW, Y-CW, and ZW performed the statistical analyses. S-JZ and DC drafted the manuscript. All authors have read and approved the final manuscript.

## Conflict of Interest

The authors declare that the research was conducted in the absence of any commercial or financial relationships that could be construed as a potential conflict of interest.

## Publisher’s Note

All claims expressed in this article are solely those of the authors and do not necessarily represent those of their affiliated organizations, or those of the publisher, the editors and the reviewers. Any product that may be evaluated in this article, or claim that may be made by its manufacturer, is not guaranteed or endorsed by the publisher.

## Abbreviations

MRI: magnetic resonance imaging
ECV: extracellular volume fraction
MOLLI: modified Look-Locker inversion-recovery
ROI: region of interest
CmyB: cardiomyocyte breadth
SD: standard deviation
SPSS: Statistical Package for Social Sciences software.
